# Current Advancements in Serum Protein Biomarkers for Hepatitis B Virus‐Associated Hepatocyte Remodeling and Hepatocellular Carcinoma

**DOI:** 10.1002/iid3.70171

**Published:** 2025-04-07

**Authors:** Adane Adugna, Gashaw Azanaw Amare, Mohammed Jemal

**Affiliations:** ^1^ Medical Laboratory Sciences College of Health Sciences Debre Markos University Debre Markos Ethiopia; ^2^ Department of Biomedical Sciences, School of Medicine Debre Markos University Debre Markos Ethiopia

**Keywords:** HBV, HCC, hepatocyte remodeling, serum protein biomarkers

## Abstract

**Background:**

Hepatitis B virus (HBV)‐related liver cancer is the third most common cause of cancer‐related death globally. Hepatocyte remodeling, also known as hepatocyte transformation and immortalization, and hepatocellular carcinoma (HCC), are brought on by persistent inflammation caused by HBV in the host hepatocytes. One of the main concerns in the perspective of HBV‐induced hepatocyte remodeling and liver cancer is accurately identifying cancer stages to maximize early screening and detection. Biological signatures have a significant impact on solving this problem.

**Objective:**

This review article aimed to discuss the novel serum protein biomarkers for HBV‐induced hepatocyte remodeling and HCC.

**Methods:**

The information was collected from various peer‐reviewed journals through electronic searches utilizing various search engines, including PubMed, Google Scholar, HINARI, and Cochrane Library from 2017 to 2024. Keywords for searches included “serum protein biomarkers in HBV‐HCC,” “blood‐based biomarkers in HBV‐HCC,” and “viral biomarkers for HBV‐HCC.”

**Results:**

Recently, novel protein signatures have been discovered for the early diagnosis, treatment, and prognosis of HBV‐induced hepatic cell remodeling and HCC from proteomic data sets. We have discussed the recent literature on the clinical utility of the protein signatures for the diagnosis and forecasting of HBV‐associated hepatocyte remodeling and HCC, including golgi protein 73 (GP73), glypican‐3 (GPC3), midkine (MDK), des‐γ‐carboxy‐prothrombin (DCP), von Willebrand factor (vWF), pentraxin 3 (PTX3), pseudouridine synthases 7 (PUSs 7), squamous cell carcinoma antigen (SCCA), and osteopontin (OPN).

**Conclusion:**

All these protein markers also exhibit the survival of HBV‐related HCC patients, the proliferation, migration, antiapoptosis, mitogenesis, transformation, and angiogenesis of HBV‐infected hepatocytes.

## 1 Introduction

Worldwide, hepatocellular carcinoma (HCC) caused by the hepatitis B virus (HBV) accounts for around 90% of instances of primary liver cancer. Liver cancer caused by HBV is the second most common cause of death from cancer [1]. These days, HCC is on the rise and could soon account for one million cases annually [2]. Patients with chronic HBV infection had a nearly 100‐fold higher chance of developing HCC, according to prospective cohort studies [3, 4].

HCC may arise from persistent hepatocyte inflammation caused by an HBV infection. Hepatocyte damage and persistent inflammation might result from a prolonged immunological response to HBV infection in the liver [5]. This never‐ending cycle of inflammation, regeneration, and cell damage can cause genetic alterations and mutations in the liver cells, which raises the possibility of developing HBV‐HCC [6]. The transition from chronic HBV infection to HCC involves an intricate interplay between viral components, host immunological responses, and environmental effects [7].

Hepatocyte remodeling describes the anatomical and functional alterations in liver cells, usually due to liver illness or persistent HBV infection. These alterations may affect hepatocytes’ interactions with the immune system in the instance of HBV‐HCC [8]. There is proof that prolonged viral replication and inflammation in chronic hepatitis B infection can change hepatocyte signaling networks and gene expression patterns [9].

In addition, some of the immediate causes of hepatocyte remodeling and HCC are HBV‐encoded proteins’ transcriptional activation of differentiation‐regulating genes, HBV DNA integration to the hepatocyte's genome, viral mutations, and genomic instability, cellular death, stimulation of signaling networks that cause the liver cell malignancy, and DNA repair [10].

During HBV‐related hepatocyte transformations, signals from hepatic stellate cells are coupled with extracellular matrix remodeling and the proliferation of cell types resembling myofibroblasts. HBV‐HCC formation is a convoluted process involving connections across multiple genes and stages. Together, several mechanisms that promote cancer accelerate the disease's transition from inflammation to carcinogenesis [11]. There are indications that the HBV virus's altered protein products hasten the development of hepatocyte remodeling and hepatic cancer. Accurate assessment of HBV‐induced hepatocyte remodeling and prediction of HCC biomarkers aid in the early identification of HBV‐HCC and reduce mortality [12].

Current clinical uses of imaging methods like magnetic resonance imaging (MRI), computed tomography (CT) scans, and ultrasound are principally used for the diagnosis of HBV‐HCC [13]. These techniques are useful for identifying liver tumors and determining their location and size. The diagnosis of HCC can also be aided by blood tests that evaluate the levels of particular biomarkers, such as AFP [14]. The early diagnosis and treatment of HCC have greatly improved via these diagnostic techniques. Improving patient outcomes requires prompt intervention and treatment, which they make possible [15].

However, these methods have poor sensitivity, specificity, and accuracy. For instance, the overall sensitivity of MRI, CT, and ultrasound to HCC is only 62%, 48%, and 46%, respectively [16]. Hence, there is a need for increased sensitivity, specificity, and accuracy in the diagnosis of HBV‐HCC. Thus, the serum protein biomarkers are being investigated to overcome these problems. Moreover, various types of proteomic signatures have been investigated to indicate HBV‐associated hepatocyte remodeling and HCC. This review will summarize recent advances in the role of those signatures in HBV‐induced hepatocyte remodeling and HCC.

## 2 Methods

The information was collected from various peer‐reviewed journals through electronic searches utilizing various search engines, including PubMed, Google Scholar, HINARI, and Cochrane Library from 2017 to 2024. Primary studies and meta‐analyses were included in this review article. Keywords for searches included “serum protein biomarkers in HBV‐HCC,” blood‐based biomarkers in HBV‐HCC,” and “viral biomarkers for HBV‐HCC.” The studies involved were limited to the English language. On the other hand, papers written in languages other than English, papers published before 2017, and review articles were excluded from this review.

## 3 Overview of the Immunosurveillance of HBV‐HCC and Hepatocyte Remodeling

Immunosurveillance, as it relates to HBV‐HCC, is the immune system's tracking and identification of liver cells (hepatocytes) that have become malignant due to HBV infection. The immune system is essential in identifying these aberrant cells and making an effort to get rid of them [17]. On the other hand, chronic HBV infection in HBV‐HCC patients may result in immunological suppression or fatigue, thereby undermining the efficacy of immunosurveillance. This may contribute to the initiation and spread of HCC by enabling malignant hepatocytes to avoid immune system recognition and multiply [18].

It is crucial to comprehend the role that immunosurveillance plays in HBV infection to devise methods of boosting immune responses against malignant cells, which could result in better therapies for this kind of liver cancer [19]. Immunosurveillance in HBV‐HCC refers to the immune system's capacity to identify and attack hepatocytes that have become malignant due to long‐term HBV infection [20]. Moreover, T cells are essential for immunosurveillance processes because they can identify aberrant antigens transported by malignant cells. On the other hand, chronic exposure to inflammatory signals and viral antigens may lead to T‐cell depletion or malfunction in the hepatic microenvironment [21]. Immunosurveillance against HBV‐infected hepatocytes, which have undergone oncogenic transformation, may be hampered by T‐cell malfunction. In the HBV‐HCC, apoptosis can eliminate infected hepatocytes, reducing viral replication and preventing the spread of damaged cells. However, HBV has developed strategies to evade apoptosis, which can prolong infection and promote tumor growth [22].

The balance between tumor growth and immune responses is referred to as equilibrium. In the case of HBV‐HCC, a state of balance is necessary to regulate viral replication and prevent excessive inflammation that could cause tissue damage. Immune cells such as T cells and natural killer (NK) cells are essential for identifying and eliminating infected hepatocytes [23, 24]. This equilibrium is maintained by a well‐regulated immune response, which allows for effective viral control without causing significant damage to hepatocytes. On the other hand, the proliferation of immune cells in response to HBV infection and the subsequent emergence of HCC are significant processes [25].

**Figure 1:**
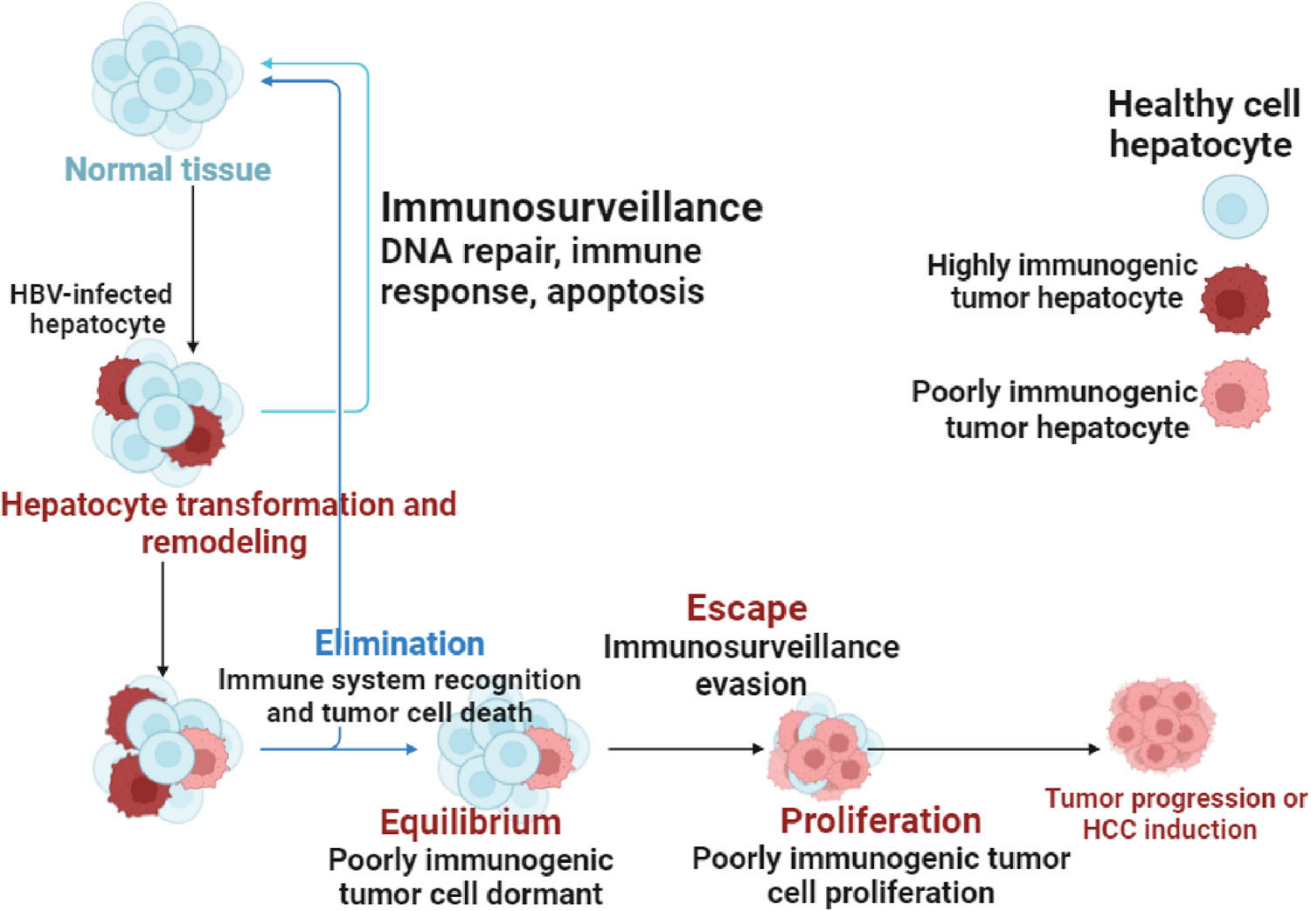
Immunosurveillance in HBV‐HCC and hepatocyte transformation.

The expansion of specialized immune cells, particularly cytotoxic T lymphocytes (CTLs), is crucial for targeting and eliminating diseased or malignant hepatocytes. However, unchecked proliferation can lead to liver damage and chronic inflammation, both of which may accelerate the development of HCC [26]. In addition, maintaining genomic stability requires DNA repair mechanisms, especially in hepatocytes that are frequently exposed to HBV infection. Genomic instability and mutations can result from HBV's integration into the host genome. To repair these damages and prevent the accumulation of mutations that may lead to cancer, DNA repair processes such as homologous recombination and nucleotide excision repair are activated [27]. Moreover, tumor‐induced changes to the extracellular matrix and architecture of the liver can produce physical barriers that obstruct efficient immune responses to HCC [28] (Figure 1).

## 4 Overview of the Role of Immune Cells in the Tumor Microenvironment of HBV‐HCC and Hepatocyte Remodeling

Immune cells are essential for controlling the hepatocyte remodeling and tumor microenvironment during HBV‐HCC [29]. Chronic HBV infection can result in a complicated interaction between growth factors, cytokines, and immune cells in the liver, which eventually aids in the development of tumors as well as immune evasion [30]. In particular, vascular endothelial growth factor is in charge of angiogenesis, which is necessary for the growth of HCC. By promoting the development of new blood vessels essential for tumor survival and proliferation, VEGF overexpression in HBV‐HCC is associated with increased tumor growth and metastasis [31].

Elevated levels of circulating VEGF have been identified in patients with HCC, and these levels correlate with increased tumor microvessel density and poorer prognosis. Studies have consistently shown that high VEGF levels, both in tissue and serum, are significant predictors of poor overall survival and disease‐free survival in HCC patients. Additionally, transforming growth factor‐beta (TGF‐β) plays a critical role as a regulator of liver fibrosis and immunosuppression. It is also involved in activating fibroblasts and promoting the synthesis of extracellular matrix components, contributing to the progression of liver disease and tumor development [32, 33]. In addition, cell migration and invasion are promoted by the over activation of the TGF‐β signaling pathway [34].

Furthermore, pro‐inflammatory cytokines such as interleukin‐1 beta (IL‐1β) can induce angiogenesis and exacerbate hepatocyte damage within the tumor microenvironment [35]. While M‐CSF promotes the differentiation of macrophages from monocytes into tissue‐resident macrophages involved in tissue repair within HCC tumors, IL‐10 suppresses effector T‐cell responses in the tumor microenvironment, thereby limiting antitumor immunity [36, 37]. Moreover, how these immune cells interact with stromal elements like fibroblasts shapes the HBV‐HCC microenvironment by regulating inflammatory responses and the promotion or suppression of angiogenesis, which in turn affects the disease's progression or regression to a great extent.

Both the immunological response to HBV and the development of HCC are influenced by TNF‐α. Its role as a pro‐inflammatory cytokine highlights the delicate balance between the potential for liver damage that can lead to cancer. Moreover, the formation and progression of HBV‐HCC heavily depend on epithelial‐mesenchymal transition, which is triggered by viral proteins such as HBX. These proteins alter cellular signaling pathways and promote a more aggressive tumor phenotype [38]. In addition, the formation and progression of HBV‐HCC are significantly influenced by cancer‐associated fibroblasts due to their roles in immune evasion, regulation of the tumor microenvironment, stimulation of tumor‐initiating cells, and heterogeneity [39].

**Figure 2:**
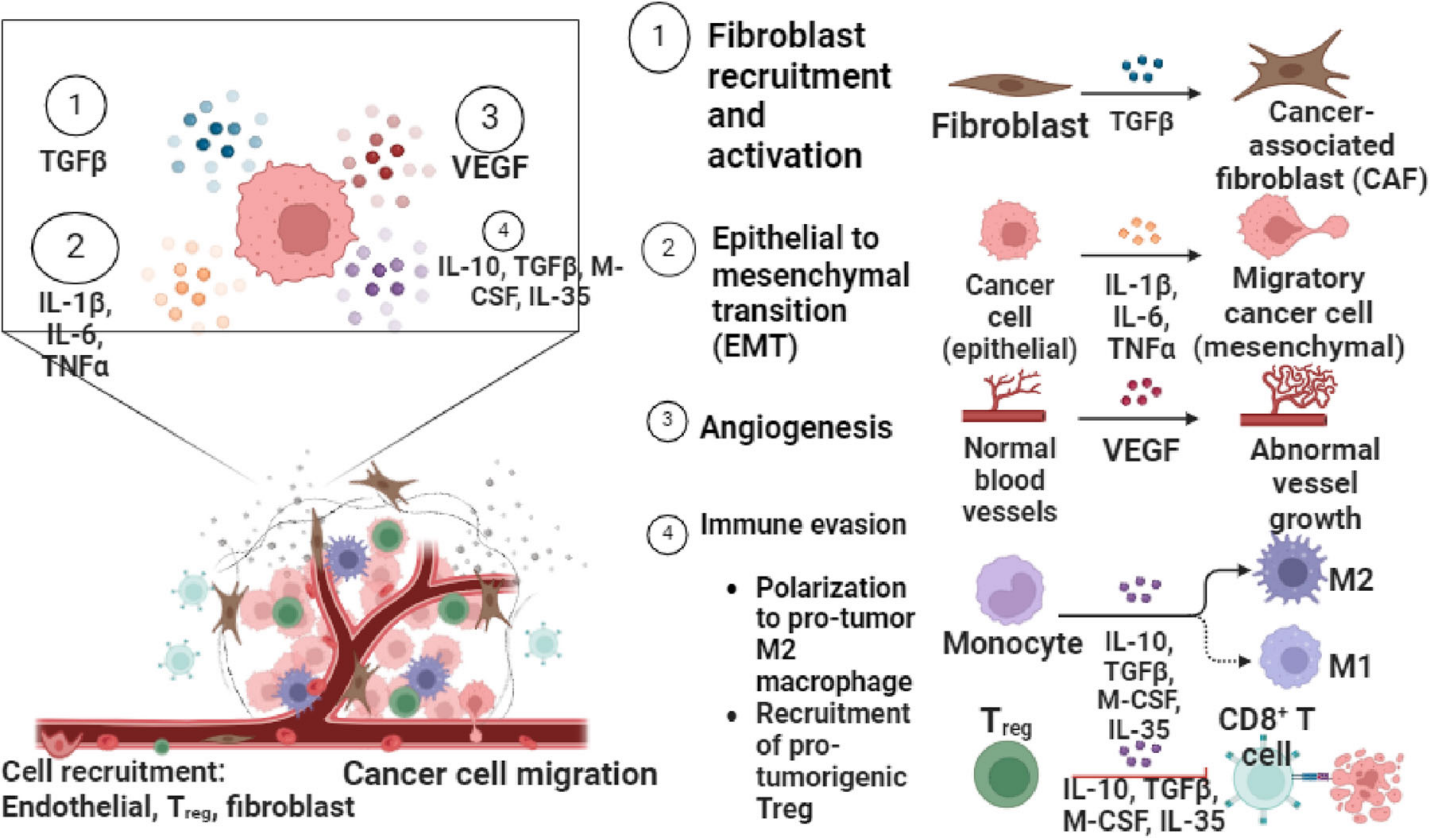
Tumor microenvironment in HBV‐HCC and hepatocyte transformation.

In the context of HBV‐HCC, interleukin‐35 (IL‐35) plays a critical role in modulating the immune response, particularly through its effects on regulatory T cells (Tregs). Tregs release IL‐35, which contributes to immune evasion by promoting T cell exhaustion, including that of CD8+ T cells [40, 41]. As a result, this leads to decreased antitumor immunity. Meanwhile, the balance between M1 and M2 macrophages is important; M1 macrophages are related to pro‐inflammatory responses that can inhibit tumor progression, whereas M2 macrophages promote an immunosuppressive environment conducive to tumor development [42, 43]. Furthermore, CD8+ T cells, which are crucial for targeting HBV‐infected hepatocytes, often become exhausted due to persistent HBV infection and the presence of Tregs and M2 macrophages [24, 44] (Figure 2).

## 5 Overview on the Role of Protein Markers in HBV‐Related Hepatocyte Remodeling

Protein markers play a multifaceted role in HBV‐related hepatocyte remodeling, offering crucial insights into the infection's status, the extent of liver damage, and the risk of progression to more severe liver diseases [45]. Understanding these markers enhances diagnostic strategies, facilitates monitoring of disease progression, and aids in the development of targeted therapies for more effective management of HBV‐related liver disease [46]. Through proteomic analysis or other applicable approaches, unique protein markers associated with hepatic remodeling during HBV infection should be identified and verified [47].

The functional roles of these protein indicators are significant, as they are involved in key biological processes such as cellular proliferation, apoptosis (programmed cell death), inflammation, fibrosis (the formation of excess connective tissue), and other mechanisms that contribute to the remodeling of hepatocytes (liver cells) [48]. The effect of these proteins on hepatocyte remodeling can be further elucidated by using molecular biology approaches such as gene silencing or overexpression. Significant evidence of these protein markers’ functional involvement can be obtained by correlating their expression levels or activities with clinical parameters, including liver function tests or disease progression in patients infected with HBV [49].

## 6 Protein Glycosylation in HBV‐HCC

Protein glycosylation has been identified as a critical factor in the development and progression of HCC in the context of HBV infection, exhibiting both tumor‐promoting and tumor‐suppressing effects. Abnormal glycosylation patterns, such as altered N‐glycosylation and O‐glycosylation, are frequently observed during HBV‐HCC progression and are implicated in the malignant transformation of hepatocytes [50]. These modifications can promote tumor growth by enhancing protumorigenic signaling pathways, facilitating immune evasion, increasing metastatic potential, and contributing to resistance against therapies. For example, dysregulated glycosylation can alter cell‐cell interactions and adhesive properties, which are crucial for tumor invasion and metastasis [51].

Conversely, certain glycosylation changes may confer tumor‐suppressive effects. For instance, increased α2,6‐sialylation has been linked to reduced metastatic capabilities in some HCC cell lines. This duality highlights the importance of the specific context of glycosylation alterations in determining their overall impact on HCC outcomes [52]. Moreover, studies have shown that HBV itself can manipulate glycosylation processes to enhance its replication and immune evasion while contributing to carcinogenesis. For example, N‐linked glycosylation in HBV surface proteins has been associated with virion assembly, immune escape, and carcinogenic processes. Understanding the complex dynamics of protein glycosylation not only provides insights into the mechanisms underlying HBV‐HCC but also offers potential avenues for developing novel diagnostic markers and therapeutic strategies targeting glycan modifications [53].

Moreover, O‐GlcNAcylation stabilizes oncogenic proteins, such as YTHDF2 and SLC35B4, enhancing their activity and driving tumorigenesis in HBV‐HCC. Elevated O‐GlcNAcylation levels promote the stabilization of mRNA transcripts for cell cycle regulators, contributing to HCC development. For example, O‐GlcNAcylation of proteins, such as YTHDF2, has been shown to enhance their stability and oncogenic activity, thereby promoting tumorigenesis in HBV‐HCC [54]. Similarly, SLC35B4‐mediated O‐GlcNAcylation stabilizes c‐MYC protein, which is critical for HCC proliferation and migration [55]. Targeting OGT, the enzyme responsible for O‐GlcNAcylation, has shown promise as a therapeutic strategy; its inhibition via compounds like OSMI‐1 effectively suppresses HBV‐HCC progression in preclinical models. Understanding the role of O‐GlcNAcylation in HBV‐HCC underscores its potential as a target for novel therapeutic interventions [56].

In addition, protein glycosylation often undergoes significant changes as cancer cells proliferate in HBV‐HCC. Specifically, the initiation, development, and metastasis of tumors have been shown to be facilitated by alterations in glycan macro‐heterogeneity and microheterogeneity in glycoproteins primarily produced by the liver [57].

Furthermore, modifications on the glycosylation pattern can affect how proteins related to the genesis of cancer function and signaling [58]. In addition, changes in cellular glycosylation give cells adaptive advantages during tumor progression [59]. Tumor progression may be impacted by inhibition of enzymes involved in the manufacture of aberrant glycans linked to malignancy [60].

## 7 Selection Criteria for Appropriate Protein Signatures for HBV‐HCC

Several criteria must be carefully considered while choosing protein biomarkers for HBV‐HCC to guarantee their efficacy and dependability for therapeutic, prognostic, and diagnostic uses [61]. Selecting protein biomarkers for HBV‐HCC requires meeting several important criteria, including specificity, which determines whether HBV‐HCC is present; sensitivity, which determines whether early‐stage HCC or minimal residual disease after treatment is detected; predictive value for prognosis and treatment response; stability, which links expression levels to relevant clinical parameters like tumor stage and patient survival outcomes; and reproducibility, which can be achieved across different laboratories and experimental settings using standardized protocols and assay kits [62, 63].

When assessing protein markers in HBV‐HCC, diagnostic accuracy is crucial, and the area under the curve (AUC) is a useful metric to evaluate overall performance. Cohort sizes in studies assessing protein markers vary often, although bigger cohorts typically yield more accurate diagnostic accuracy estimates [64].

For instance, a recent large‐scale cohort of 500 patients with HBV‐HCC and 500 healthy controls demonstrated high diagnosis accuracy in a study looking into a protein marker for HBV‐HCC. The protein marker showed an important discriminatory ability in separating HCC from nonmalignant conditions with an AUC of 85%, and sensitivity and specificity of ≥ 80%. The detection method employed, the enzyme‐linked immunosorbent assay (ELISA), ensures applicability in medical settings. These results demonstrate the potential utility of the protein biomarker as a diagnostic tool for HBV‐HCC surveillance and screening initiatives [65].

## 8 Key Serum Protein Biomarkers for HBV‐Associated Hepatocyte Remodeling and HCC

### 8.1 Alpha‐Fetoprotein (AFP)

AFP is one of the most commonly used biomarkers for diagnosing HCC in patients with chronic HBV infection. Moreover, AFP is most commonly recognized as an oncofetal glycoprotein that has been used in conjunction with imaging modalities, such as ultrasound, to improve the diagnosis of HCC [66]. However, its effectiveness is limited by issues related to sensitivity and specificity, which can be influenced by patient characteristics and the varying cut‐off values employed for differential diagnosis. Recent studies have identified several limitations related with AFP, including its relatively low sensitivity and high false‐positive rate, particularly in cases of HBV‐HCC. For example, a study demonstrated that an AFP cutoff of 400 ng/mL yielded a sensitivity of only 51.3% and a specificity of 87.8% for detecting HBV‐HCC. Furthermore, combining AFP with other biomarkers, such as des‐gamma‐carboxyprothrombin, has been shown to enhance diagnostic performance, achieving a combination sensitivity of 55.6% and a specificity of 95.6% [67, 68]. Despite its established role, the effectiveness of AFP is complicated by its elevation in a wide range of liver diseases, highlighting the need for ongoing evaluation of additional biomarkers and combination strategies to improve early detection and management of HCC in patients with chronic HBV infection [69].

### 8.2 Golgi Protein (GP73)

Transmembrane glycoprotein GP73 is a component of the Golgi apparatus. GP73 is expressed by hepatocytes in HBV‐HCC. Hepatic inflammatory activity may be the cause of elevated GP73 levels. The amount of GP73 is elevated in patients with HCC and may be a target for therapy. Its sensitivity and specificity for predicting HBV‐HCC are 69% and 75%, respectively, which is far better than AFP's (62% and 25%) detection of early HCC in the same group [70]. The role of GP73 in HCC carcinogenesis is not well discussed, although GP73 has been the subject of numerous research focusing on its use as a marker for early HCC detection [71].

A deeper comprehension of GP73's function in HCC may offer a novel therapeutic target for the disease, considering the low response to the HCC treatments that are now available. According to immunohistochemistry investigations, GP73 protein expression in the liver increased steadily with pathologic progression during persistent HBV infection [72].

In other words, the disease severity of chronic HBV infections was positively linked with serum GP73 concentrations. Since variations in the severity of liver injury are strongly correlated with changes in serum GP73 levels, GP73 may be a valuable novel biomarker for inflammatory liver injury and may be useful for tracking the prognosis of chronic HBV infection. Furthermore, the trend of GP73 fluctuation in chronic liver disease may suggest that serum GP73 monitoring is useful for identifying cirrhosis in populations with chronic HBV infection [73]. GP73 expression is also noticeably increased in HBV‐HCC tissues. In vitro, GP73 expression in primary human hepatocytes and hepatoma cells may be induced by ectopic expression of the HBV gene. In hepatoma cells, GP73 encourages HCC. The host's innate immune responses are suppressed in hepatocytes by the ectopic expression of HBV genes, which enhances GP73 expression [74].

### 8.3 Glypican‐3 (GPC3)

A member of the glypicans family, GPC3 attaches to the cell surface using glycosylated phosphatidyl inositol. GPC‐3 is recognized as a significant marker for HCC due to its elevated expression in the liver during carcinogenesis and its absence in normal liver tissue [75]. For the identification of HBV‐HCC, GPC3 can serve as a marker, demonstrating an AUC of 0.817, a sensitivity of 91.3%, and a specificity of 60.0%. Furthermore, aberrant GPC‐3 expression serves as an independent prognostic factor for the survival of patients with HBV‐HCC [76].

In other words, GPC‐3 is typically overexpressed in malignant tissues while remaining undetectable in healthy liver cells and benign liver lesions. This unique expression pattern enhances its utility as both a diagnostic biomarker and a potential therapeutic target in HCC management. Studies have shown that high levels of GPC‐3 correlate with aggressive tumor behavior and poorer patient outcomes, including reduced overall survival and disease‐free survival rates. Through several cytokines, the GPC3 protein is closely linked to the body's growth and development. Plasma GPC3 may be a useful marker for identifying people who would benefit from immunotherapies that target GPC3 [77, 78]. Hence, GPC‐3 can be targeted for a novel immunotherapy strategy that can specify cell‐mediated destruction of neoplastic cells while sparing normal liver tissue [79].

Furthermore, a meta‐analysis comparing the diagnostic performance of GPC‐3 and AFP revealed significant findings regarding their sensitivity, specificity, and AUC. The pooled sensitivity and specificity were 55% and 58% for GPC‐3, 54% and 83% for AFP, and 85% and 79% for the combination of GPC‐3 and AFP. The AUC values were 0.7793 for GPC‐3, 0.7867 for AFP, and a notably higher 0.9366 for the combined markers. These results indicate that while GPC‐3 has comparable sensitivity to AFP, its specificity is lower; however, the combination of GPC‐3 and AFP significantly enhances diagnostic accuracy, as demonstrated by the higher AUC for the combined marker compared to either marker alone [80].

Various immunotherapeutic protocols targeting GPC3 have been developed, including the use of humanized anti‐GPC3 cytotoxic antibodies, treatment with peptide/DNA vaccines, and immunotoxin therapies [81]. As it may be essential for cell proliferation, metastasis, and colonization as well as mediating oncogenesis and oncogenic signaling pathways, GPC‐3 is also a viable target molecule for HBV‐related HCC gene therapy [82].

### 8.4 Midkine (MDK)

Heparin‐binding growth factor/cytokine called MDK has several uses and is involved in many physiological processes like viability, cell movement, and other cell processes [83]. Rarely produced by normal tissues, MDK is markedly increased in inflammatory disorders and human malignant tumors, particularly HCC‐HBV. Many types of solid tumors, including HCC, exhibit proliferation, migration, antiapoptosis, mitogenesis, transformation, and angiogenesis, and studies have shown that MDK plays a significant role in these processes. The antiapoptotic factor MDK promotes the survival of tumor cells. Additionally, MDK suppresses anoikis to encourage metastasis [84].

Anti‐MDK methods may be applied with success in the treatment of HCC, according to some evidence. Additionally, MDK has been suggested as a biomarker in the prognosis and diagnosis of early HCC particularly in the serum negative AFP patients because of the high expression in HCC. The sensitivity, specificity, and AUC of MDK and AFP for identifying HCC were pooled using a random‐effects model in the prior systematic review and meta‐analysis. The following are the summary values for early‐stage HCC detection using MDK and AFP: sensitivity, 83.5 versus 44.4%; specificity, 81.7 versus 84.8%; and AUC, 0.87 versus 0.52. In patients with HCC, this marker may be used as a prognostic indicator [85, 86].

### 8.5 Des‐γ‐Carboxy‐Prothrombin (DCP)

DCP is increasingly recognized as a critical serum indicator for the diagnosis of HCC‐HBV. In addition, DCP levels have been shown to associate with more aggressive tumor behavior and poorer clinical outcomes, making it not only a diagnostic tool but also a prognostic indicator [87]. After curative radiofrequency ablation (RFA), postablation serum levels of DCP serve as a crucial biomarker for predicting survival and recurrence in patients with HBV‐HCC. The higher DCP levels following ablation are associated with worse prognosis and an increased likelihood of cancer recurrence. Specifically, individuals with elevated DCP levels after RFA tend to experience poorer overall survival and recurrence‐free survival rates compared to those with lower levels [88].

DCP, also known as prothrombin induced by vitamin K absence or antagonist‐II (PIVKA‐II), is significant in clinical practice. PIVKA‐II levels are elevated in HCC patients due to impaired prothrombin carboxylation in cancerous cells caused by a deficiency of vitamin K. This anomaly leads to the accumulation of PIVKA‐II in the bloodstream, making it a valuable marker for HCC diagnosis and surveillance. Furthermore, the high levels of PIVKA‑II showed a more aggressive tumor phenotype, and they may also offer new insights into the process underlying the spread of HCC cells and make it easier to create new therapeutic approaches for patients with early HCC [89]. PIVKA‐II outperformed AFP in the retrospective cohort study in terms of accuracy for diagnosing overall recurrent HBV‐HCC (AUC: 0.883 vs. 0.672; *p* < 0.0001) [90].

### 8.6 Von Willebrand Factor (vWF)

VWF is found in platelets and endothelial cells and is essential for hemostasis and tissue damage. A study revealed that VWF's distinct functional structure and capacity could facilitate the metastasis of cancer. An earlier study found that when compared to the HCC group, the VWF plasma concentration was significantly lower in CHB (chronic hepatitis B) and healthy controls [91]. The overexpression of this biomarker was detected from the liver biopsy of patients with CHBV infection [92]. VWF is linked to hepatic spare capacity and HCC and has been linked to angiogenesis and apoptosis. VWF is a biomarker with the potential to be helpful in the diagnosis and prognosis of aggressive HBV infection‐related HCC. Hepatic spare capacity and VWF antigen are linked because vWF‐Ag is elevated in HCC patients. This pathology is connected to angiogenesis [93]. Disintegrin and metalloproteinase with thrombospondin type 1 motif, member 13 (DAMTS13) regulates the size and activity of the VWF, and its absence can result in an expansion of the VWF [94].

Previous research suggests that vWF‐Ag inhibition therapy should be considered as part of the treatment plan for patients with chronic hepatic inflammation. This approach may assist clinicians in streamlining monitoring methods [95]. Plasma vWF levels and in vitro scores can assess the degree of liver cirrhosis decompensation and the progression of liver disease in patients with HBV infection. They can also predict the development of various complications following liver cirrhosis decompensation and serve as a guide for early intervention strategies [96].

### 8.7 Pentraxin 3 (PTX3)

The relation between PTX3 and HCC may be affected by its function in the immune response. For instance, interleukin (IL)‐1 and tumor necrosis factor (TNF)‐α can generate PTX3 expression during HBV‐HCC and hepatocyte immortalization [97]. Moreover, PTX3 is involved in the control of biological processes connected to HBV‐HCC, including metastasis, angiogenesis, and proliferation. Studies on HCC have revealed that PTX3 may speed up the disease's development. Patients with HCC who have elevated PTX3 expression in tumor tissues have a bad prognosis. Serum PTX3 levels are related to the onset of HCC in HBV infection and are useful in the diagnosis of HCC, even AFP‐negative HCC [98, 99, 100].

Depending on the study and technique, several diagnostic accuracy metrics such as AUC, sensitivity, and specificity may change. Data from patients with cirrhosis, HCC, and chronic hepatitis caused by HBV were evaluated, as well as data from healthy controls. By using an immunoassay to test the serum PTX3 concentration, it was found that patients with HBV had levels that were significantly greater than those of healthy controls, and patients with HCC had levels that were higher than those of those with chronic hepatitis or cirrhosis [101]. A lower overall survival time was seen in HCC patients with high serum PTX3 levels (PTX3 > 9.25 ng/mL) compared to HCC patients with low serum PTX3 levels (PTX3 9.25 ng/mL). Patients with HCC connected to HBV are prognostically predicted using serum PTX3 levels as a biomarker [101].

Elevated levels of PTX3 represent an independent risk factor for HBV‐HCC. In patients with HCC, PTX3 levels appear to correlate with tumor differentiation. The significance of serum PTX3 levels lies in their ability to effectively differentiate HCC from chronic hepatitis, cirrhosis, and chronic HBV infection without HCC. Moreover, measuring PTX3 levels could provide substantial diagnostic value for HBV‐HCC, particularly in cases that are AFP‐negative and at early stages of the HCC [100]. With an AUC of 0.948, the combination of AFP and PTX3 enhanced the capacity to distinguish between early HCC and chronic HBV infection. In preclinical models and certain cancers, PTX3 is an extrinsic oncosuppressor by controlling complement‐driven macrophage‐mediated tumor growth and adjusting cancer‐related inflammation [98, 100].

### 8.8 Pseudouridine Synthases (PUSs)

PUSs are emerging as a novel therapeutic target for HBV‐HCC. Specifically, PUSs act as a glioma growth regulator that can be targeted, with higher expression levels associated with poorer patient survival outcomes. Furthermore, treatment with PUS7 inhibitors has been shown to slow tumor growth and extend the lifespan of mice with tumors [102]. Studies have shown that many PUS genes, such as DKC1, PUS1, and PUS7, are significantly upregulated in HBV‐HCC tissues and are related with poor patient prognosis. These enzymes are involved in posttranscriptional RNA modifications, influencing key cellular processes such as metabolism, cell cycle regulation, and immune response. Elevated PUS expression has been identified as a potential diagnostic indicator for HBV‐HCC and is implicated in disease progression [102, 103]. Additionally, their role in critical metabolic pathways highlights the potential of targeting PUSs as a new therapeutic approach for HBV‐HCC treatment [103].

### 8.9 Squamous Cell Carcinoma Antigen (SCCA) and Antibodies Against SCCA/SERPINB3

Recent study has revealed that systemic concentrations of squamous cell carcinoma antigen‐immunoglobulin M (SCCA‐IgM) increase over time and may indicate the development of cirrhosis in individuals with chronic hepatitis. SCCA has been shown to prevent apoptosis in cancer cells in HBV‐HCC, and its measurement has proven valuable in identifying individuals with HCC. Elevated SCCA‐IgM levels can effectively distinguish HCC from chronic hepatitis, cirrhosis, and chronic HBV infection without HCC, highlighting its potential role in monitoring disease progression and guiding clinical management [104].

The ability of AFP to diagnose HCC is enhanced by SCCA by up to 90%. There is strong proof that SCCA activity rises gradually throughout the development of liver cancer, from chronic hepatitis to dysplastic tumors to HCC. SERPINB3, also known as squamous cell carcinoma antigen‐1 (SCCA1), is a serine protease inhibitor that plays a dual role in cellular processes. While it protects cells from reactive oxygen species, its persistent expression can contribute to the development of HBV‐HCC. SERPINB3 promotes oncogenesis by inhibiting apoptosis, facilitating the transition from epithelial to mesenchymal phenotypes, reducing desmosomal junctions, and enhancing cell proliferation and invasion. This multifaceted involvement in cancer progression underscores its potential as a therapeutic target, particularly in HBV‐HCC, where its elevated levels may indicate disease severity and poor prognosis [105]. The most significant specific response for human SerpinB3 is demonstrated by anti‐P#5 antibodies, which are generated by targeting the reactive helix of SerpinB3 [106].

HepG2 cells inducing SerpinB3 were used to test the biological efficacy of each immunoglobulin formulation. Anti‐P#5 antibody decreased the spread of cells by 75% and multiplication by 12%, whereas the other immunoglobulin formulations produced negligible outcomes [107]. These results suggest that SerpinB3's responsive region is crucial for the intrusion properties this serpin induces, and it may develop into a potential druggable domain. SB3, which is generated by damaged or inflamed hepatic cells may function as an inflammatory mediator in HBV‐HCC, accelerating the course of the illness. A single serum SCCA‐IgM assay aids in identifying hepatic cirrhosis patients who are more likely to have HCC and die from it [108].

### 8.10 Osteopontin (OPN)

OPN is a highly modified integrin‐binding glycoprotein found abundantly in various cells and tissues. It plays a significant role in cancer metastasis, cellular metabolism, tissue healing, and carcinogenesis. In particular, tumor organs such as the liver have been shown to overexpress OPN [109]. Moreover, increased circulating OPN concentrations in HBV‐HCC have been linked to intrahepatic metastasis and the early stages of tumor development. OPN is frequently used as a biomarker to predict the progression of HCC, particularly in its initial stages. Its elevated levels are related with poor prognosis among individuals with HCC, suggesting that OPN may play a significant role in tumor marker‐driven cancer progression [110].

OPN levels in the HBsAg‐positive HCC population are significantly higher than those in HBsAg‐negative individuals with other malignancies and in the unaffected control group. The absence of OPN is related with increased overall survival, reduced chronic inflammation, and a lower incidence of poorly differentiated tumors in HCC. Furthermore, OPN outperformed AFP in terms of diagnostic sensitivity, specificity, and overall accuracy for HCC patients compared to the cirrhotic group (97%, 70%, and 84% at the 90 ng/mL cut‐off value for OPN vs. 90%, 63%, and 77% at the 5.5 ng/mL cut‐off value for AFP) [111, 112] (Table 1). The major protein signatures with their respective diagnostic accuracy are summarized in Table 1 below.

**Table 1:** List of the main serum protein biomarkers with their respective diagnostic accuracy in different stages of HBV‐HCC.

Proteomic signatures	Detection techniques	Patient groups (HBV‐HCC stages)	Sensitivity (%)	Specificity (%)	Accuracy (%)	AUC	Cut‐off values (ng/mL)	Ref.
GP73	ELISA	Early stage	76.3	80.1	79.6	0.78	> 5	[71]
GP73 + AFP	ELISA	All stages	89.2	85.2	96	0.82	6.5	[74]
GP73 + DKK‐1	ELISA	Advanced stage	97.4	93.1	94.5	0.86	7.4	[71]
GPC3	IHC	Recurrence stage	55	58	56.6	0.46	6.1	[80]
GPC3 + AFP	Western blot analysis	Recurrence stage	85	79	82.2	0.94	6.8	[80]
MDK	IHC	Early stage	93	85	89.3	0.95	> 5	[86]
DCP	RIA	Recurrence stage	59.9–71	89.4–93	74.4–82.2	0.76–0.91	8.1	[87, 88, 113]
vWF	Protein microarrays	Early stage	79.5	92.3	88.6–90.9	0.721–0.964	6.3	[93, 95, 96]
PTX3	Western blot analysis	Intermediate stage	85.1	89.3	87.3	0.93	9.2	[100]
PTX3 + AFP	IHC	Early stage	89.7	87.4	88.4	0.96	9.1	[101]
SCCA	IHC	Cirrhotic patients	59	76	67.3	0.78–0.80	6.7	[108]
ESERPINB3/SCCA‐IGM	ELISA	Intermediate stage	85	92	89	0.88	> 5	[114]
SCCA‐IGM + AFP	CLIA	Intermediate stage	88	94	91.3	0.90	> 5	[115]
OPN	CLIA	Early‐stage	97	70	84	0.86	> 5	[116]

Abbreviations: AUC, area under the curve; CLIA, chemiluminescent immunoassay; DKK‐1, dickkopf‐1; ELISA, enzyme linked immunosorbent assay; IHC, Immunohistochemistry; ng/mL, nanograms per milliliter; Ref, reference; RIA, radioimmunoassay.

## 9 Limitations of the Present Review

Selection bias could affect this review and cause the evidence to be presented in an unbalanced way. It also does not have a methodical strategy for finding and combining evidence, which leaves gaps in the literature's coverage and raises the possibility of missing important studies. Furthermore, there may be significant variations in the breadth and quality of the included research, which could result in inconsistent findings from the review. Furthermore, by focusing only on published literature and ignoring unpublished or gray literature, this review may be more vulnerable to publication bias due to its lack of quantitative data analysis. Due to the absence of a consistent technique for data synthesis, the conclusions drawn from this research may not necessarily apply to other populations. Subsequently, there is a greater chance that subjective interpretation will affect the conclusions made from the information because it mostly relies on qualitative interpretation rather than just quantitative analysis.

## 10 Conclusions and Future Directions

Even with the increasing understanding of the pathogenesis of HBV‐HCC, HBV‐induced hepatocyte remodeling and HCC remain challenging to identify and treat in the early stages. Evaluation is still needed for newly developed protein signatures (indicators) specific to liver malignancies that ensure better patient identification and longevity. Our review summarized the types and clinical applications of protein signatures for the prompt identification and diagnosis of HBV‐induced hepatocyte transformations and hepatocarcinogenesis. On the other hand, these signatures served as diagnostic, therapeutic, and prognostic targets for HBV‐caused hepatocyte remodeling and HCC.

Future developments in proteomic biomarkers for HBV‐HCC are probably going to concentrate on enhancing these biomarkers’ sensitivity, specificity, and accuracy to facilitate tailored treatment plans and aid in early diagnosis, prognosis, and treatment response monitoring. In general, greater sensitivity and specificity, standardization/validation efforts, integration with other omics data types, noninvasive detection techniques, personalized medicine approaches, and tracking disease progression/treatment response are likely to be the main focuses of serum protein biomarkers for HBV‐HCC in the future. These developments could lead to better patient outcomes as well as more effective resource allocation within the healthcare system.

## Author Contributions

**Adane Adugna:** conceptualization, data curation, formal analysis, investigation, methodology, software, validation, writing – original draft, writing – review and editing. **Gashaw Azanaw Amare:** conceptualization, methodology, software, supervision, validation, visualization, writing – original draft, writing – review and editing. **Mohammed Jemal:** conceptualization, data curation, formal analysis, supervision, validation, visualization, writing – original draft, writing – review and editing.

## Ethics Statement

The authors have nothing to report.

## Conflicts of Interest

The authors declare no conflicts of interest.

## Data Availability

The data presented in this manuscript have been available on the hand of corresponding author for reasonable request.

## References

[1] C. Cassinotto , E. Nogue , Q. Durand , et al., “Life Expectancy of Patients With Hepatocellular Carcinoma According to the Upfront Treatment: A Nationwide Analysis,” Diagnostic and Interventional Imaging no. 104 4 (2023): 192–199. 36682959 10.1016/j.diii.2023.01.002

[2] J. M. Llovet , R. Montal , D. Sia , and R. S. Finn , “Molecular Therapies and Precision Medicine for Hepatocellular Carcinoma,” Nature Reviews Clinical Oncology 15, no. 10 (2018): 599–616. 10.1038/s41571-018-0073-4 PMC12452113 30061739

[3] S. P. Kaur , A. Talat , and H. Karimi‐Sari , “Hepatocellular Carcinoma in Hepatitis B Virus‐Infected Patients and the Role of Hepatitis B Surface Antigen (HBsAg),” Journal of Clinical Medicine 11 no. 4 (2022): 1126. 35207397 10.3390/jcm11041126 PMC8878376

[4] G. E. M. Rizzo , G. Cabibbo , and A. Craxì , “Hepatitis B Virus‐Associated Hepatocellular Carcinoma,” Viruses 14, no. 5 (2022): 986. 35632728 10.3390/v14050986 PMC9146458

[5] H. J. Cho and J. Y. Cheong , “Role of Immune Cells in Patients With Hepatitis B Virus‐Related Hepatocellular Carcinoma,” International Journal of Molecular Sciences 22, no. 15 (2021): 8011. 34360777 10.3390/ijms22158011 PMC8348470

[6] A. Khanam and J. V. Chua , “Immunopathology of Chronic Hepatitis B,” Infection: Role of Innate and Adaptive Immune Response in Disease Progression 22, no. 11 (2021): 5497. 10.3390/ijms22115497 PMC8197097 34071064

[7] R. Nevola , D. Beccia , V. Rosato , et al., “HBV Infection and Host Interactions: The Role in Viral Persistence and Oncogenesis,” International Journal of Molecular Sciences 24, no. 8 (2023): 7651. 37108816 10.3390/ijms24087651 PMC10145402

[8] T. Kanda , R. Sasaki‐Tanaka , and S. Terai , “Inflammation of the Liver, HCC Development and HCC Establishment,” Hepatology International 18, no. 4 (2024): 1090–1092. 38951373 10.1007/s12072-024-10707-0

[9] M. G. Refolo and C. Messa , “Inflammatory Mechanisms of HCC,” Development 12 (2020): 3. 10.3390/cancers12030641 PMC7139884 32164265

[10] W. S. Mason , A. R. Jilbert , and S. Litwin , “Hepatitis B Virus DNA Integration and Clonal Expansion of Hepatocytes in the Chronically Infected Liver,” Viruses 13, no. 2 (2021): 210. 33573130 10.3390/v13020210 PMC7911963

[11] I. H. Sarker , “Deep Learning: A Comprehensive Overview on Techniques, Taxonomy, Applications and Research Directions,” SN Computer Science 2, no. 6 (2021): 420. 34426802 10.1007/s42979-021-00815-1 PMC8372231

[12] Y. Huang , Y. Pi , K. Ma , et al., “Deep Learning for Patient‐Specific Quality Assurance: Predicting Gamma Passing Rates for IMRT Based on Delivery Fluence Informed by Log Files,” Technology in Cancer Research & Treatment 21 (2022): 15330338221104881. 35726209 10.1177/15330338221104881 PMC9218492

[13] C. Song , M. Huang , X. Zhou , et al., “Prediction of Immunocyte Infiltration and Prognosis in Postoperative Hepatitis B Virus‐Related Hepatocellular Carcinoma Patients Using Magnetic Resonance Imaging,” Gastroenterology Report 12 (2024): goae009. 38415224 10.1093/gastro/goae009 PMC10898339

[14] K. Tzartzeva , J. Obi , N. E. Rich , et al., “Surveillance Imaging and Alpha Fetoprotein for Early Detection of Hepatocellular Carcinoma in Patients With Cirrhosis: A Meta‐Analysis,” Gastroenterology 154, no. 6 (2018): 1706–1718.e1. 29425931 10.1053/j.gastro.2018.01.064 PMC5927818

[15] P. R. Galle and F. Foerster , “Biology and Significance of Alpha‐Fetoprotein in Hepatocellular Carcinoma,” Liver International 39 no. 12 (2019): 2214–2229. 31436873 10.1111/liv.14223

[16] X. Xu , X. Zhou , B. Xiao , et al., “Glutathione‐Responsive Magnetic Nanoparticles for Highly Sensitive Diagnosis of Liver Metastases,” Nano Letters 21 no. 5 (2021): 2199–2206. 33600181 10.1021/acs.nanolett.0c04967

[17] G. Yang , P. Wan , Y. Zhang , et al., “Innate Immunity, Inflammation, and Intervention in HBV Infection,” Viruses 14 no. 10 (2022): 2275. 36298831 10.3390/v14102275 PMC9609328

[18] T. Quaye and P. W. Narkwa , “Immunosurveillance and Molecular Detection of Hepatitis B Virus Infection Amongst Vaccinated Children in the West Gonja District in Savanna Region of Ghana,” PLoS One 16 no. 9 (2021): e0257103. 34534234 10.1371/journal.pone.0257103 PMC8448355

[19] L. L. Boeijen , R. C. Hoogeveen , A. Boonstra , and G. M. Lauer , “Hepatitis B Virus Infection and the Immune Response: The Big Questions,” Best Practice & Research Clinical Gastroenterology 31, no. 3 (2017): 265–272. 28774408 10.1016/j.bpg.2017.05.003

[20] M. Y. W. Zaki , A. M. Fathi , S. Samir , et al., “Innate and Adaptive Immunopathogeneses in Viral Hepatitis; Crucial Determinants of Hepatocellular Carcinoma,” Cancers 14, no. 5 (2022): 1255. 35267563 10.3390/cancers14051255 PMC8909759

[21] F. Meng , J. Zhao , A. T. Tan , et al., “Immunotherapy of HBV‐Related Advanced Hepatocellular Carcinoma With Short‐Term HBV‐Specific TCR Expressed T Cells: Results of Dose Escalation, Phase I Trial,” Hepatology International 15 (2021): 1402–1412. 34850325 10.1007/s12072-021-10250-2 PMC8651587

[22] Y. Jiang , Q. Han , H. Zhao , and J. Zhang , “The Mechanisms of HBV‐Induced Hepatocellular Carcinoma,” Journal of Hepatocellular Carcinoma 8 (2021): 435–450. 34046368 10.2147/JHC.S307962 PMC8147889

[23] R. Sun , J. Li , X. Lin , et al., “Peripheral Immune Characteristics of Hepatitis B Virus‐Related Hepatocellular Carcinoma,” Frontiers in Immunology 14 (2023): 1079495. 37077908 10.3389/fimmu.2023.1079495 PMC10106696

[24] M. N. Khan , B. Mao , J. Hu , et al., “Tumor‐Associated Macrophages and CD8+ T Cells: Dual Players in the Pathogenesis of HBV‐Related HCC,” Frontiers in Immunology 15 (2024): 1472430. 39450177 10.3389/fimmu.2024.1472430 PMC11499146

[25] J. He , R. Miao , Y. Chen , H. Wang , and M. Liu , “The Dual Role of Regulatory T Cells in Hepatitis B Virus Infection and Related Hepatocellular Carcinoma,” Immunology 171, no. 4 (2024): 445–463. 38093705 10.1111/imm.13738

[26] L. Galasso , L. Cerrito , V. Maccauro , et al., “Inflammatory Response in the Pathogenesis and Treatment of Hepatocellular Carcinoma: A Double‐Edged Weapon,” International Journal of Molecular Sciences 25, no. 13 (2024): 7191. 39000296 10.3390/ijms25137191 PMC11241080

[27] L. Yang , T. Zou , Y. Chen , et al., “Hepatitis B Virus X Protein Mediated Epigenetic Alterations in the Pathogenesis of Hepatocellular Carcinoma,” Hepatology International 16, no. 4 (2022): 741–754. 35648301 10.1007/s12072-022-10351-6

[28] B. Yue , Y. Gao , Y. Hu , M. Zhan , Y. Wu , and L. Lu , “Harnessing CD8+ T Cell Dynamics in Hepatitis B Virus‐Associated Liver Diseases: Insights, Therapies and Future Directions,” Clinical and Translational Medicine 14, no. 7 (2024): e1731. 38935536 10.1002/ctm2.1731 PMC11210506

[29] Z. Li , Z. Zhang , L. Fang , et al., “Tumor Microenvironment Composition and Related Therapy in Hepatocellular Carcinoma,” Journal of Hepatocellular Carcinoma 10 (2023): 2083–2099. 38022729 10.2147/JHC.S436962 PMC10676104

[30] J. Wu , M. Han , J. Li , X. Yang , and D. Yang , “Immunopathogenesis of HBV Infection,” Hepatitis B Virus Infection: Molecular Virology to Antiviral Drugs 20, no. 39 (2020): 71–107. 10.1007/978-981-13-9151-4_4 31741334

[31] C. Yao , S. Wu , J. Kong , et al., “Angiogenesis in Hepatocellular Carcinoma: Mechanisms and Anti‐Angiogenic Therapies,” Cancer Biology & Medicine 20, no. 1 (2023): 25–43. 36647777 10.20892/j.issn.2095-3941.2022.0449 PMC9843448

[32] E. Pinto , F. Pelizzaro , R. Cardin , et al., “HIF‐1α and VEGF as Prognostic Biomarkers in Hepatocellular Carcinoma Patients Treated With Transarterial Chemoembolization,” Digestive and Liver Disease 56, no. 5 (2024): 872–879. 37783655 10.1016/j.dld.2023.09.019

[33] J. E. Yoo and J. H. Nahm , “The Dual Role of Transforming Growth Factor‐Beta Signatures in Human B Viral Multistep Hepatocarcinogenesis: Early and Late Responsive Genes,” Journal of Liver Cancer 22 no. 2 (2022): 115–124. 37383409 10.17998/jlc.2022.04.20 PMC10035736

[34] H. Wang , P. Wang , M. Xu , et al., “Distinct Functions of Transforming Growth Factor‐β Signaling in c‐MYC Driven Hepatocellular Carcinoma Initiation and Progression,” Cell Death & Disease 12 no. 2 (2021): 200. 33608500 10.1038/s41419-021-03488-z PMC7895828

[35] J. Fischer , S. Long , and E. Koukoulioti , “Association of Common Polymorphisms in the Interleukin‐1 Beta Gene With Hepatocellular Carcinoma in Caucasian Patients With Chronic Hepatitis B,” Pathogens 12 no. 1 (2022): 54. 36678401 10.3390/pathogens12010054 PMC9861021

[36] Y. Akazawa , H. Kono , M. Hara , et al., “M‐CSF Receptor Antagonists Inhibit the Initiation and Progression of Hepatocellular Carcinoma in Mice,” Anticancer Research 39, no. 9 (2019): 4787–4794. 31519580 10.21873/anticanres.13663

[37] K. Heim and Sagar , “Attenuated Effector T Cells Are Linked to Control of Chronic HBV Infection,” Nature Immunology 25 no. 9 (2024): 1650–1662. 39198634 10.1038/s41590-024-01928-4 PMC11362014

[38] S. Gurzu , L. Kobori , D. Fodor , and I. Jung , “Epithelial Mesenchymal and Endothelial Mesenchymal Transitions in Hepatocellular Carcinoma: A Review,” BioMed Research International 2019 (2019): 1–12. 10.1155/2019/2962580 PMC6855070 31781608

[39] L. Yu , N. Shen , Y. Shi , et al., “Characterization of Cancer‐Related Fibroblasts (CAF) in Hepatocellular Carcinoma and Construction of CAF‐Based Risk Signature Based on Single‐Cell RNA‐Seq and Bulk RNA‐Seq Data,” Frontiers in Immunology 13 (2022): 1009789. 36211448 10.3389/fimmu.2022.1009789 PMC9537943

[40] M. Hu , J. Zhang , J. Zhu , H. Fang , and J. Liu , “Prognostic Signifcance of IL35 Expression in Human Hepatocellular Carcinoma,” International Journal of Clinical and Experimental Pathology 11, no. 9 (2018): 4695–4702. 31949870 PMC6962944

[41] X. Liu , H. Ren , H. Guo , W. Wang , and N. Zhao , “Interleukin‐35 Has a Tumor‐Promoting Role in Hepatocellular Carcinoma,” Clinical and Experimental Immunology 203 no. 2 (2021): 219–229. 33030251 10.1111/cei.13535 PMC7806415

[42] K. Arvanitakis and T. Koletsa , “Tumor‐Associated Macrophages in Hepatocellular Carcinoma Pathogenesis, Prognosis and Therapy,” Cancers (Basel) 14 no. 1 (2022): 226. 35008390 10.3390/cancers14010226 PMC8749970

[43] Y. Huang , W. Ge , J. Zhou , B. Gao , X. Qian , and W. Wang , “The Role of Tumor Associated Macrophages in Hepatocellular Carcinoma,” Journal of Cancer 12, no. 5 (2021): 1284–1294. 33531974 10.7150/jca.51346 PMC7847664

[44] J. Li , H. Chen , L. Bai , and H. Tang , “Identification of CD8+ T‐Cell Exhaustion Signatures for Prognosis in HBV‐Related Hepatocellular Carcinoma Patients by Integrated Analysis of Single‐Cell and Bulk RNA‐Sequencing,” BMC Cancer 24, no. 1 (2024): 53. 38200408 10.1186/s12885-023-11804-3 PMC10777580

[45] A. Kramvis , K.‐M. Chang , M. Dandri , et al., “A Roadmap for Serum Biomarkers for Hepatitis B Virus: Current Status and Future Outlook,” Nature Reviews Gastroenterology & Hepatology 19, no. 11 (2022): 727–745. 35859026 10.1038/s41575-022-00649-z PMC9298709

[46] N. Varghese , A. Majeed , S. Nyalakonda , T. Boortalary , and D. Halegoua‐DeMarzio , “Review of Related Factors for Persistent Risk of Hepatitis B Virus‐Associated Hepatocellular Carcinoma,” Cancers 16 no. 4 (2024): 777. 38398168 10.3390/cancers16040777 PMC10887172

[47] F. Zhao , X. Xie , X. Tan , et al., “The Functions of Hepatitis B Virus Encoding Proteins: Viral Persistence and Liver Pathogenesis,” Frontiers in Immunology 12 (2021): 691766. 34456908 10.3389/fimmu.2021.691766 PMC8387624

[48] K. Dalal , P. Khorate , B. Dalal , et al., “Differentially Expressed Serum Host Proteins in Hepatitis B and C Viral Infections,” VirusDisease 29 (2018): 468–477. 30539049 10.1007/s13337-018-0484-y PMC6261891

[49] Z. Xiang , J. Li , D. Lu , X. Wei , and X. Xu , “Advances in Multi‐Omics Research on Viral Hepatitis,” Frontiers in Microbiology 13 (2022): 987324. 36118247 10.3389/fmicb.2022.987324 PMC9478034

[50] M. Kohansal‐Nodehi , M. Swiatek‐de Lange , K. Kroeniger , et al., “Discovery of a Haptoglobin Glycopeptides Biomarker Panel for Early Diagnosis of Hepatocellular Carcinoma,” Frontiers in Oncology 13 (2023): 1213898. 37920152 10.3389/fonc.2023.1213898 PMC10619681

[51] R. P. Aryal , M. Noel , J. Zeng , et al., “Cosmc Regulates O‐Glycan Extension in Murine Hepatocytes,” Glycobiology 34 no. 10 (2024): cwae069. 39216105 10.1093/glycob/cwae069 PMC11398974

[52] B. Cao , M. Duan , Y. Xing , et al., “O‐GlcNAc Transferase Activates Stem‐Like Cell Potential in Hepatocarcinoma Through O‐GlcNAcylation of Eukaryotic Initiation Factor 4E,” Journal of Cellular and Molecular Medicine 23, no. 4 (2019): 2384–2398. 30677218 10.1111/jcmm.14043 PMC6433694

[53] A. DelaCourt , A. Black , and P. Angel , “N‐Glycosylation Patterns Correlate With Hepatocellular Carcinoma Genetic Subtypes,” Molecular Cancer Research 19 no. 11 (2021): 1868–1877. 34380744 10.1158/1541-7786.MCR-21-0348 PMC8802325

[54] Y. Yang , Y. Yan , J. Yin , and N. Tang , “O‐GlcNAcylation of YTHDF2 Promotes HBV‐Related Hepatocellular Carcinoma Progression in an N(6)‐Methyladenosine‐Dependent Manner,” Signal Transduction and Targeted Therapy 8 no. 1 (2023): 63. 36765030 10.1038/s41392-023-01316-8 PMC9918532

[55] T. Jiang , J. Yang , H. Yang , et al., “SLC35B4 Stabilizes c‐MYC Protein by O‐GlcNAcylation in HCC,” Frontiers in Pharmacology 13 (2022): 851089. 35308201 10.3389/fphar.2022.851089 PMC8924407

[56] L. Zhong , D. Liao , M. Zhang , et al., “YTHDF2 Suppresses Cell Proliferation and Growth via Destabilizing the EGFR mRNA in Hepatocellular Carcinoma,” Cancer Letters 442 (2019): 252–261. 30423408 10.1016/j.canlet.2018.11.006

[57] T. H. Su , C. Y. Peng , T. C. Tseng , et al., “Serum Mac‐2‐Binding Protein Glycosylation Isomer at Virological Remission Predicts Hepatocellular Carcinoma and Death in Chronic Hepatitis B‐Related Cirrhosis,” Journal of Infectious Diseases 221, no. 4 (2020): 589–597. 31574141 10.1093/infdis/jiz496

[58] D. Thomas , A. K. Rathinavel , and P. Radhakrishnan , “Altered Glycosylation in Cancer: A Promising Target for Biomarkers and Therapeutics,” Biochimica et Biophysica Acta (BBA)—Reviews on Cancer 1875, no. 1 (2021): 188464. 33157161 10.1016/j.bbcan.2020.188464 PMC7855613

[59] G. Siracusano , M. Tagliamonte , L. Buonaguro , and L. Lopalco , “Cell Surface Proteins in Hepatocellular Carcinoma: From Bench to Bedside,” Vaccines 8, no. 1 (2020): 41. 31991677 10.3390/vaccines8010041 PMC7157713

[60] T. Jun , Y. C. Hsu , S. Ogawa , et al., “Mac‐2 Binding Protein Glycosylation Isomer as a Hepatocellular Carcinoma Marker in Patients With Chronic Hepatitis B or C Infection,” Hepatology Communications 3 no. 4 (2019): 493–503. 30976740 10.1002/hep4.1321 PMC6442699

[61] U. Landegren and M. Hammond , “Cancer Diagnostics Based on Plasma Protein Biomarkers: Hard Times but Great Expectations,” Molecular Oncology 15, no. 6 (2021): 1715–1726. 33012111 10.1002/1878-0261.12809 PMC8169444

[62] J. Hartl , F. Kurth , K. Kappert , et al., “Quantitative Protein Biomarker Panels: A Path to Improved Clinical Practice Through Proteomics,” EMBO Molecular Medicine 15, no. 4 (2023): 16061. 10.15252/emmm.202216061 PMC10086577 36939029

[63] E. C. Paver and A. L. Morey , “Biomarkers and Biomarker Validation: A Pathologist's Guide to Getting It Right,” Pathology 56, no. 2 (2024): 147–157. 38195376 10.1016/j.pathol.2023.11.002

[64] F. Piñero , M. Dirchwolf , and M. G. Pessôa , “Biomarkers in Hepatocellular Carcinoma: Diagnosis, Prognosis and Treatment Response Assessment,” Cells 9, no. 6 (2020): 1370. 32492896 10.3390/cells9061370 PMC7349517

[65] L. Y. Mak , “Disease Modifiers and Novel Markers in HBV‐Related HCC,” Journal of Liver Cancer 9 (2024). 10.17998/jlc.2024.08.03 PMC11449577 39099070

[66] J. Sun , Y. Zhao , L. Qin , et al., “Metabolomic Profiles for HBV Related Hepatocellular Carcinoma Including Alpha‐Fetoproteins Positive and Negative Subtypes,” Frontiers in Oncology 9 (2019): 1069. 31681602 10.3389/fonc.2019.01069 PMC6803550

[67] A. M. Aliyu , A. B. Olokoba , M. O. Bojuwoye , and K. C. Okonkwo , “The Combined Diagnostic Accuracy of Serum Alpha Fetoprotein and Des‐Gamma Carboxyprothrombin in Hepatocellular Carcinoma Among Chronic Liver Disease Patients in Ilorin,” Open Journal of Gastroenterology 11, no. 12 (2021): 255–274.

[68] I. M. Khan , D. Gjuka , J. Jiao , et al., “A Novel Biomarker Panel for the Early Detection and Risk Assessment of Hepatocellular Carcinoma in Patients With Cirrhosis,” Cancer Prevention Research 14, no. 6 (2021): 667–674. 33685927 10.1158/1940-6207.CAPR-20-0600 PMC8225562

[69] C.‐W. Lee , H.‐I. Tsai , W.‐C. Lee , et al., “Normal Alpha‐Fetoprotein Hepatocellular Carcinoma: Are They Really Normal?,” Journal of Clinical Medicine 8, no. 10 (2019): 1736. 31635078 10.3390/jcm8101736 PMC6832124

[70] Y.‐T. Chan , C. Zhang , J. Wu , et al., “Biomarkers for Diagnosis and Therapeutic Options in Hepatocellular Carcinoma,” Molecular Cancer 23, no. 1 (2024): 189. 39242496 10.1186/s12943-024-02101-z PMC11378508

[71] Y. Wang and Y. J. Y. Wan , “Golgi Protein 73, Hepatocellular Carcinoma and Other Types of Cancers,” Liver Research 4, no. 4 (2020): 161–167. 33343966 10.1016/j.livres.2020.09.003 PMC7743997

[72] M. Wei , Z. Xu , X. Pan , et al., “Serum GP73—An Additional Biochemical Marker for Liver Inflammation in Chronic HBV Infected Patients With Normal or Slightly Raised ALT,” Scientific Reports 9, no. 1 (2019): 1170. 30718535 10.1038/s41598-018-36480-3 PMC6362062

[73] M. K. Shaker , F. M. Attia , A. A. Hassan , M. M. Shedid , M. A. Aboelmagd , and A. S. Faisal , “Evaluation of Golgi Protein 73 (GP73) as a Potential Biomarkers for Hepatocellular Carcinoma,” Clinical Laboratory 9, no. 8 (2020). 10.7754/Clin.Lab.2020.190911 32776730

[74] M. Wu , Z. Liu , X. Li , A. Zhang , and N. Li , “Dynamic Changes in Serum Markers and Their Utility in the Early Diagnosis of All Stages of Hepatitis B‐Associated Hepatocellular Carcinoma,” OncoTargets and Therapy 13 (2020): 827–840. 32095079 10.2147/OTT.S229835 PMC6995291

[75] B. Batbaatar , U. Gurbadam , O. Tuvshinsaikhan , et al., “Evaluation of Glypican‑3 in Patients With Hepatocellular Carcinoma,” Molecular and Clinical Oncology 22, no. 1 (2024): 1. 39534882 10.3892/mco.2024.2796 PMC11552472

[76] N. Li , T. Dong , P. Wang , Q. Li , and F. Nie , “Predicting Glypican‐3 Expression in Hepatocellular Carcinoma: A Comprehensive Analysis Using Combined Contrast‐Enhanced Ultrasound and Clinical Factors,” Clinical Hemorheology and Microcirculation 85, no. 4 (2023): 407–420. 37638421 10.3233/CH-231912

[77] B. Moudi , Z. Heidari , H. Mahmoudzadeh‐Sagheb , et al., “Concomitant Use of Heat‐Shock Protein 70, Glutamine Synthetase and Glypican‐3 Is Useful in Diagnosis of HBV‐Related Hepatocellular Carcinoma With Higher Specificity and Sensitivity,” European Journal of Histochemistry: EJH 62, no. 1 (2018): 2859. 29569872 10.4081/ejh.2018.2859 PMC5806503

[78] P. Rochigneux , B. Chanez , B. De Rauglaudre , E. Mitry , C. Chabannon , and M. Gilabert , “Adoptive Cell Therapy in Hepatocellular Carcinoma: Biological Rationale and First Results in Early Phase Clinical Trials,” Cancers 13, no. 2 (2021): 271. 33450845 10.3390/cancers13020271 PMC7828372

[79] B. Varghese , N. Darayan , C. Rastellini , L. Cicalese , and M. Montalbano , “New Immunological Approaches in Hepatocellular Carcinoma: Glypican‐3 (GPC‐3) Opportunities and Challenges,” Journal of Cancer Therapy 11, no. 11 (2020): 647–659.

[80] D. Xu , C. Su , L. Sun , Y. Gao , and Y. Li , “Performance of Serum Glypican 3 in Diagnosis of Hepatocellular Carcinoma: A Meta‐Analysis,” Annals of Hepatology 18, no. 1 (2019): 58–67. 31113610 10.5604/01.3001.0012.7863

[81] F. Zhou , W. Shang , X. Yu , and J. Tian , “Glypican‐3: A Promising Biomarker for Hepatocellular Carcinoma Diagnosis and Treatment,” Medicinal Research Reviews 38, no. 2 (2018): 741–767. 28621802 10.1002/med.21455

[82] F. Rossari , S. Foti , S. Camera , M. Persano , A. Casadei‐Gardini , and M. Rimini , “Treatment Options for Advanced Hepatocellular Carcinoma: The Potential of Biologics,” Expert Opinion on Biological Therapy 24, no. 6 (2024): 455–470. 38913107 10.1080/14712598.2024.2363234

[83] P. S. Filippou , G. S. Karagiannis , and A. Constantinidou , “Midkine (MDK) Growth Factor: A Key Player in Cancer Progression and a Promising Therapeutic Target,” Oncogene 39, no. 10 (2020): 2040–2054. 31801970 10.1038/s41388-019-1124-8

[84] B. H. Zhang , B. Li , L. X. Kong , L. N. Yan , and J. Y. Yang , “Diagnostic Accuracy of Midkine on Hepatocellular Carcinoma: A Meta‐Analysis,” PLoS One 14, no. 10 (2019): 0223514. 10.1371/journal.pone.0223514 PMC6786585 31600291

[85] A. Gowhari Shabgah , F. Ezzatifar , S. Aravindhan , et al., “Shedding More Light on the Role of Midkine in Hepatocellular Carcinoma: New Perspectives on Diagnosis and Therapy,” IUBMB Life 73, no. 4 (2021): 659–669. 33625758 10.1002/iub.2458

[86] Q. Lu , J. Li , H. Cao , C. Lv , X. Wang , and S. Cao , “Comparison of Diagnostic Accuracy of Midkine and AFP for Detecting Hepatocellular Carcinoma: A Systematic Review and Meta‐Analysis,” Bioscience Reports 40, no. 3 (2020): BSR20192424. 32039435 10.1042/BSR20192424 PMC7087326

[87] J. Chen , G. Wu , and Y. Li , “Evaluation of Serum Des‐Gamma‐Carboxy Prothrombin for the Diagnosis of Hepatitis B Virus‐Related Hepatocellular Carcinoma: A Meta‐Analysis,” Disease Markers 2018 (2018): 1–10. 10.1155/2018/8906023 PMC6193331 30402170

[88] T. Song , L. Wang , R. Xin , L. Zhang , and Y. Tian , “Evaluation of Serum AFP and DCP Levels in the Diagnosis of Early‐Stage HBV‐Related HCC Under Different Backgrounds,” Journal of International Medical Research 48, no. 10 (2020): 0300060520969087. 33135527 10.1177/0300060520969087 PMC7780580

[89] X. L. Ma , J. Zhu , J. Wu , et al., “Significance of PIVKA‑II Levels for Predicting Microvascular Invasion and Tumor Cell Proliferation in Chinese Patients With Hepatitis B Virus‑Associated Hepatocellular Carcinoma,” Oncology Letters 15, no. 6 (2018): 8396–8404. 29805574 10.3892/ol.2018.8375 PMC5950517

[90] W. Zhu , W. Wang , W. Zheng , et al., “Diagnostic Performance of PIVKA‐II in Identifying Recurrent Hepatocellular Carcinoma Following Curative Resection: A Retrospective Cohort Study,” Scientific Reports 14, no. 1 (2024): 8416. 38600210 10.1038/s41598-024-59174-5 PMC11006886

[91] A. Elhence and Shalimar , “Von Willebrand Factor as a Biomarker for Liver Disease—An Update,” Journal of Clinical and Experimental Hepatology 13, no. 6 (2023): 1047–1060. 37975050 10.1016/j.jceh.2023.05.016 PMC10643510

[92] D. J. Groeneveld , L. G. Poole , and J. P. Luyendyk , “Targeting von Willebrand Factor in Liver Diseases: A Novel Therapeutic Strategy?,” Journal of Thrombosis and Haemostasis 19, no. 6 (2021): 1390–1408. 33774926 10.1111/jth.15312 PMC8582603

[93] B. Ye , Y. Shen , H. Chen , et al., “Differential Proteomic Analysis of Plasma‐Derived Exosomes as Diagnostic Biomarkers for Chronic HBV‐Related Liver Disease,” Scientific Reports 12, no. 1 (2022): 14428. 36002595 10.1038/s41598-022-13272-4 PMC9402575

[94] E. Roose and B. S. Joly , “Current and Future Perspectives on ADAMTS13 and Thrombotic Thrombocytopenic Purpura,” Hämostaseologie 40, no. 03 (2020): 322–336. 32726827 10.1055/a-1171-0473

[95] H. Takaya , H. Kawaratani , Y. Tsuji , et al., “von Willebrand Factor Is a Useful Biomarker for Liver Fibrosis and Prediction of Hepatocellular Carcinoma Development in Patients With Hepatitis B and C,” United European Gastroenterology Journal 6 no. 9 (2018): 1401–1409. 30386613 10.1177/2050640618779660 PMC6206538

[96] Y. L. Guan , D. Z. Zhang , Y. X. Yang , et al., “[The Clinical Value of von Willebrand Factor and VITRO Score in Evaluating Disease Progression in Patients With HBV Infection],” Zhonghua Gan Zang Bing Za Zhi = Zhonghua Ganzangbing Zazhi = Chinese Journal of Hepatology 30, no. 3 (2022): 309–315. 35462488 10.3760/cma.j.cn501113-20210202-00061 PMC12770295

[97] E. M. Helal , S. M. Shoeib , and S. M. Mansour , “The Potential Diagnostic Value of Serum Pentraxin‐3 in Hepatocellular Carcinoma in Egyptian Patients,” Egyptian Liver Journal 14, no. 1 (2024): 37.

[98] A. Doni , M. Stravalaci , A. Inforzato , et al., “The Long Pentraxin PTX3 as a Link Between Innate Immunity, Tissue Remodeling, and Cancer,” Frontiers in Immunology 10 (2019): 712. 31019517 10.3389/fimmu.2019.00712 PMC6459138

[99] T. Song , C. Wang , C. Guo , Q. Liu , and X. Zheng , “Pentraxin 3 Overexpression Accelerated Tumor Metastasis and Indicated Poor Prognosis in Hepatocellular Carcinoma via Driving Epithelial‐Mesenchymal Transition,” Journal of Cancer 9, no. 15 (2018): 2650–2658. 30087705 10.7150/jca.25188 PMC6072810

[100] H. Deng , X. Fan , X. Wang , et al., “Serum Pentraxin 3 as a Biomarker of Hepatocellular Carcinoma in Chronic Hepatitis B Virus Infection,” Scientific Reports 10, no. 1 (2020): 20276. 33219288 10.1038/s41598-020-77332-3 PMC7680106

[101] Q. Han , H. Deng , and X. Fan , “Increased Serum Pentraxin 3 Levels Are Associated With Poor Prognosis of Hepatitis B Virus‐Related Hepatocellular Carcinoma,” Journal of Hepatocellular Carcinoma 8 (2021): 1367–1373. 34805015 10.2147/JHC.S337936 PMC8598127

[102] Z. Jin , M. Song , J. Wang , et al., “Integrative Multiomics Evaluation Reveals the Importance of Pseudouridine Synthases in Hepatocellular Carcinoma,” Frontiers in Genetics 13 (2022): 944681. 36437949 10.3389/fgene.2022.944681 PMC9686406

[103] Y. X. Hu and L. T. Diao , “Pseudouridine Synthase 1 Promotes Hepatocellular Carcinoma Through mRNA Pseudouridylation to Enhance the Translation of Oncogenic mRNAs,” Hepatology 80 no. 5 (2024): 1058–1073. 38015993 10.1097/HEP.0000000000000702

[104] M. Cagnin , A. Biasiolo , A. Martini , et al., “Serum Squamous Cell Carcinoma Antigen‐Immunoglobulin M Complex Levels Predict Survival in Patients With Cirrhosis,” Scientific Reports 9, no. 1 (2019): 20126. 31882893 10.1038/s41598-019-56633-2 PMC6934856

[105] E. Novo , A. Cappon , G. Villano , et al., “SerpinB3 as a Pro‐Inflammatory Mediator in the Progression of Experimental Non‐Alcoholic Fatty Liver Disease,” Frontiers in Immunology 13 (2022): 910526. 35874657 10.3389/fimmu.2022.910526 PMC9304805

[106] A. Biasiolo and M. Sandre , “Epitope‐Specific Anti‐SerpinB3 Antibodies for SerpinB3 Recognition and Biological Activity Inhibition,” Biomolecules 13 no. 5 (2023): 739. 37238609 10.3390/biom13050739 PMC10216589

[107] S. Cannito , B. Foglia , G. Villano , et al., “SerpinB3 Differently Up‐Regulates Hypoxia Inducible Factors‐1α and‐2α in Hepatocellular Carcinoma: Mechanisms Revealing Novel Potential Therapeutic Targets,” Cancers 11, no. 12 (2019): 1933. 31817100 10.3390/cancers11121933 PMC6966556

[108] P. Pontisso and M. Parola , “SERPINB3 in Fibrogenic Chronic Liver Diseases and Primary Liver Cancers,” Exploration of Digestive Diseases 3 (2024): 22–41.

[109] W. Rong , Y. Zhang , L. Yang , et al., “Post‐Surgical Resection Prognostic Value of Combined OPN, MMP7, and PSG9 Plasma Biomarkers in Hepatocellular Carcinoma,” Frontiers of Medicine 13, no. 2 (2019): 250–258. 29770948 10.1007/s11684-018-0632-1

[110] M. Zhu , J. Zheng , F. Wu , et al., “OPN Is a Promising Serological Biomarker for Hepatocellular Carcinoma Diagnosis,” Journal of Medical Virology 92, no. 12 (2020): 3596–3603. 32043608 10.1002/jmv.25704

[111] H. B. Liu , Q. Y. Chen , X. Y. Wang , et al., “Infection With Hepatitis B Virus May Increase the Serum Concentrations of Osteopontin,” Intervirology 64, no. 3 (2021): 126–134. 33735879 10.1159/000513687 PMC8491474

[112] E. F. Mostafa , H. M. Eltaher , E. Nasr Eldin , and S. M. Hassany , “Diagnostic Accuracy of Plasma Osteopontin in Egyptian Hepatocellular‎ Carcinoma Patients,” Afro‐Egyptian Journal of Infectious and Endemic Diseases 13, no. 1 (2023): 3–14.

[113] Y. Chen , X. Yang , Y. Shao , et al., “Comparison of Diagnostic Performance of AFP, DCP and Two Diagnostic Models in Hepatocellular Carcinoma: A Retrospective Study,” Annals of Hepatology 28, no. 4 (2023): 101099. 37030571 10.1016/j.aohep.2023.101099

[114] M. Xing , X. Wang , R. Kirken , L. He , and J.‐Y. Zhang , “Immunodiagnostic Biomarkers for Hepatocellular Carcinoma (HCC): The First Step in Detection and Treatment,” International Journal of Molecular Sciences 22, no. 11 (2021): 6139. 34200243 10.3390/ijms22116139 PMC8201127

[115] H. Bui Huu , N. Ha Thuc , H. P. Thi Le , et al., “Characterization of SCCA‐IgM as a Biomarker of Liver Disease in an Asian Cohort of Patients,” Scandinavian Journal of Clinical and Laboratory Investigation 78, no. 3 (2018): 204–210. 29381084 10.1080/00365513.2018.1432072

[116] Y. Zhang , J. Gao , Y. Bao , et al., “Diagnostic Accuracy and Prognostic Significance of Osteopontin in Liver Cirrhosis and Hepatocellular Carcinoma: A Meta‐Analysis,” Biomarkers 27, no. 1 (2022): 13–21. 34787036 10.1080/1354750X.2021.2008009

